# Supplementary material for: Recreational Physical Activity and the Mediterranean Diet: Their Effects on Obesity-Related Body Image Dissatisfaction and Eating Disorders
Source: Healthcare (Basel). 2024 Aug 8;12(16):1579. doi: 10.3390/healthcare12161579 (PMC11353470; doi:10.3390/healthcare12161579)
Supplement: Supplementary file 1 [file healthcare-12-01579-s001.zip › healthcare-3084020-supplementary.pdf]

File S1. Descriptive Statistics

Descriptive Statistics

	Group	Gender	Mean	Std. Deviation	N
EAT-26	NBG	MEN	6,9068	5,20391	236
		WOMEN	9,1444	7,12676	277
		Total	8,1150	6,40743	513
	OBG	MEN	12,5945	6,81828	365
		WOMEN	17,4488	8,09145	127
		Total	13,8476	7,46909	492
	CG	MEN	9,2614	7,87619	88
		WOMEN	12,0283	8,59677	212
		Total	11,2167	8,47323	300
	Total	MEN	10,2206	6,96883	689
		WOMEN	11,8490	8,44658	616
		Total	10,9893	7,74160	1305
RSES Rosenberg	NBG	MEN	23,7627	2,66315	236
		WOMEN	23,0325	2,61594	277
		Total	23,3684	2,66024	513
	OBG	MEN	20,9014	3,52793	365
		WOMEN	18,7244	4,09932	127
		Total	20,3394	3,80115	492
	CG	MEN	21,9432	4,02109	88
		WOMEN	19,5472	4,63682	212
		Total	20,2500	4,59032	300
	Total	MEN	22,0145	3,57191	689
		WOMEN	20,9448	4,18867	616
		Total	21,5096	3,91044	1305
MedDietScore	NBG	MEN	41,5805	4,55952	236
		WOMEN	41,1697	4,20017	277
		Total	41,3587	4,36964	513
	OBG	MEN	30,5836	5,48492	365

		WOMEN	30,6772	5,75958	127
		Total	30,6077	5,55133	492
	CG	MEN	25,3636	5,02221	88
		WOMEN	24,8821	5,12870	212
		Total	25,0233	5,09404	300
	Total	MEN	33,6836	7,84544	689
		WOMEN	33,4010	8,80170	616
		Total	33,5502	8,30851	1305
MBSRQappearEval	NBG	MEN	3,8257	,50962	236
		WOMEN	3,7726	,66022	277
		Total	3,7970	,59571	513
	OBG	MEN	3,4939	,69610	365
		WOMEN	2,8403	,79123	127
		Total	3,3252	,77580	492
	CG	MEN	3,4838	,63683	88
		WOMEN	3,5020	,84033	212
		Total	3,4967	,78511	300
	Total	MEN	3,6063	,64922	689
		WOMEN	3,4872	,83026	616
		Total	3,5501	,74231	1305
MBSRQappearOrient	NBG	MEN	3,1451	,52263	236
		WOMEN	3,6727	,49961	277
		Total	3,4300	,57376	513
	OBG	MEN	3,1256	,61881	365
		WOMEN	3,4928	,60913	127
		Total	3,2204	,63637	492
	CG	MEN	3,1515	,46725	88
		WOMEN	3,5774	,57196	212
		Total	3,4525	,57629	300
	Total	MEN	3,1356	,56887	689
		WOMEN	3,6028	,55245	616
		Total	3,3561	,60756	1305
MBSRBASS	NBG	MEN	3,9430	,53738	236

		WOMEN	3,8476	,53416	277
		Total	3,8915	,53724	513
	OBG	MEN	3,6718	,63429	365
		WOMEN	3,2318	,72928	127
		Total	3,5583	,68695	492
	CG	MEN	3,6907	,62661	88
		WOMEN	3,6798	,76316	212
		Total	3,6830	,72476	300
	Total	MEN	3,7671	,61427	689
		WOMEN	3,6629	,70047	616
		Total	3,7179	,65818	1305
MBSROverWeigPreoc	NBG	MEN	2,4386	,57745	236
		WOMEN	2,6868	,66012	277
		Total	2,5726	,63505	513
	OBG	MEN	2,8664	,62415	365
		WOMEN	3,3839	,79856	127
		Total	3,0000	,70980	492
	CG	MEN	2,7926	,65896	88
		WOMEN	2,7724	,80969	212
		Total	2,7783	,76751	300
	Total	MEN	2,7104	,64346	689
		WOMEN	2,8600	,78991	616
		Total	2,7810	,71993	1305
MBSRQSelfClasWei	NBG	MEN	2,8136	,65003	236
		WOMEN	3,0126	,53061	277
		Total	2,9211	,59630	513
	OBG	MEN	3,5767	,65853	365
		WOMEN	4,1535	,60916	127
		Total	3,7256	,69323	492
	CG	MEN	3,5000	,86103	88
		WOMEN	3,0542	,92523	212
		Total	3,1850	,92798	300
	Total	MEN	3,3055	,77097	689

		WOMEN	3,2622	,83851	616
		Total	3,2851	,80354	1305
MBSRQFitnEval	NBG	MEN	3,7881	,63791	236
		WOMEN	3,6703	,64111	277
		Total	3,7245	,64171	513
	OBG	MEN	3,6247	,71658	365
		WOMEN	3,2651	,69685	127
		Total	3,5318	,72808	492
	CG	MEN	3,5265	,83921	88
		WOMEN	3,3679	,80143	212
		Total	3,4144	,81450	300
	Total	MEN	3,6681	,71291	689
		WOMEN	3,4827	,73108	616
		Total	3,5806	,72719	1305
MBSRQFitnOrient	NBG	MEN	3,5632	,41207	236
		WOMEN	3,3971	,58725	277
		Total	3,4735	,52029	513
	OBG	MEN	3,4040	,52820	365
		WOMEN	3,1272	,62277	127
		Total	3,3326	,56663	492
	CG	MEN	2,7858	,52412	88
		WOMEN	2,8425	,73629	212
		Total	2,8259	,68056	300
	Total	MEN	3,3796	,54527	689
		WOMEN	3,1506	,69313	616
		Total	3,2715	,62970	1305

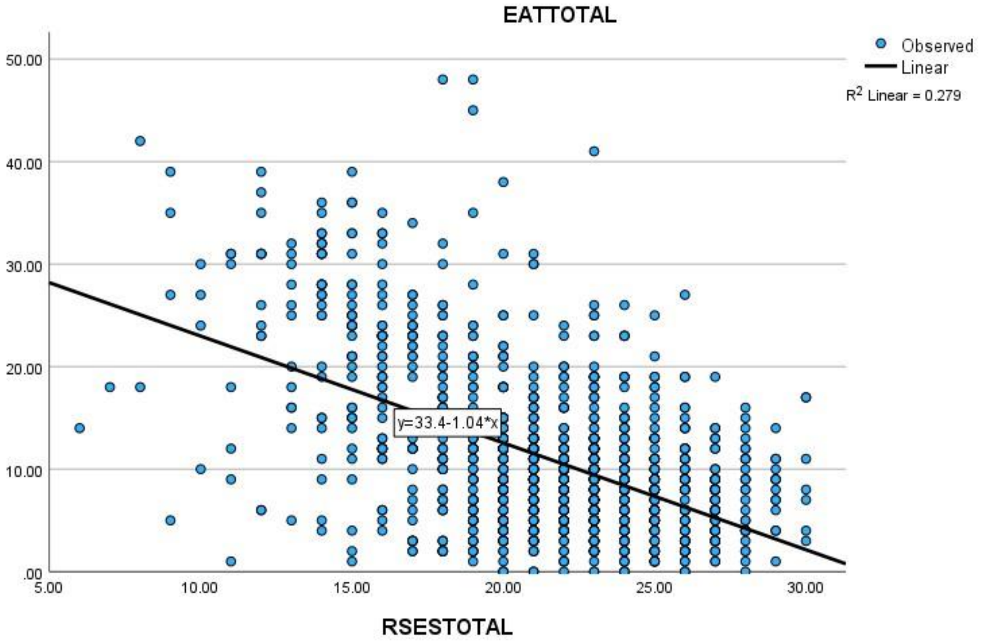
EATTOTAL
Observed
Linear
R^2 Linear = 0.279
y=33.4-1.04*x
50.00
40.00
30.00
20.00
10.00
.00
5.00
10.00
15.00
20.00
25.00
30.00
RSESTOTAL

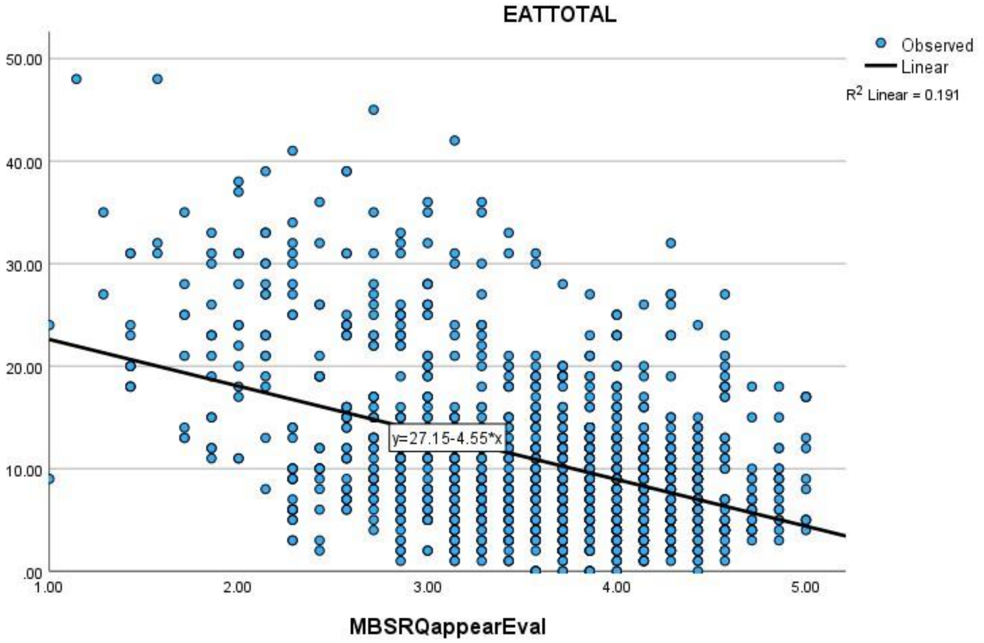
EATTOTAL
Observed
Linear
R^2 Linear = 0.191
y=27.15-4.55*x
50.00
40.00
30.00
20.00
10.00
.00
1.00
2.00
3.00
4.00
5.00
MBSRQappearEval

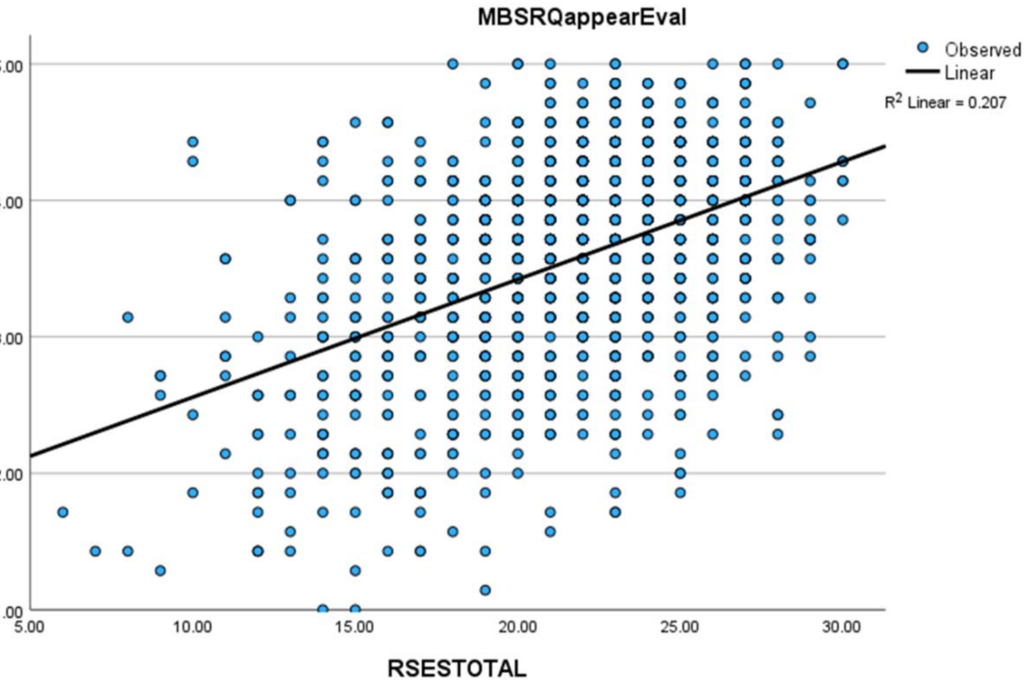
MBSRQappearEval
Observed
Linear
R2 Linear = 0.207
5.00
4.00
3.00
2.00
1.00
5.00
10.00
15.00
20.00
25.00
30.00
RSESTOTAL

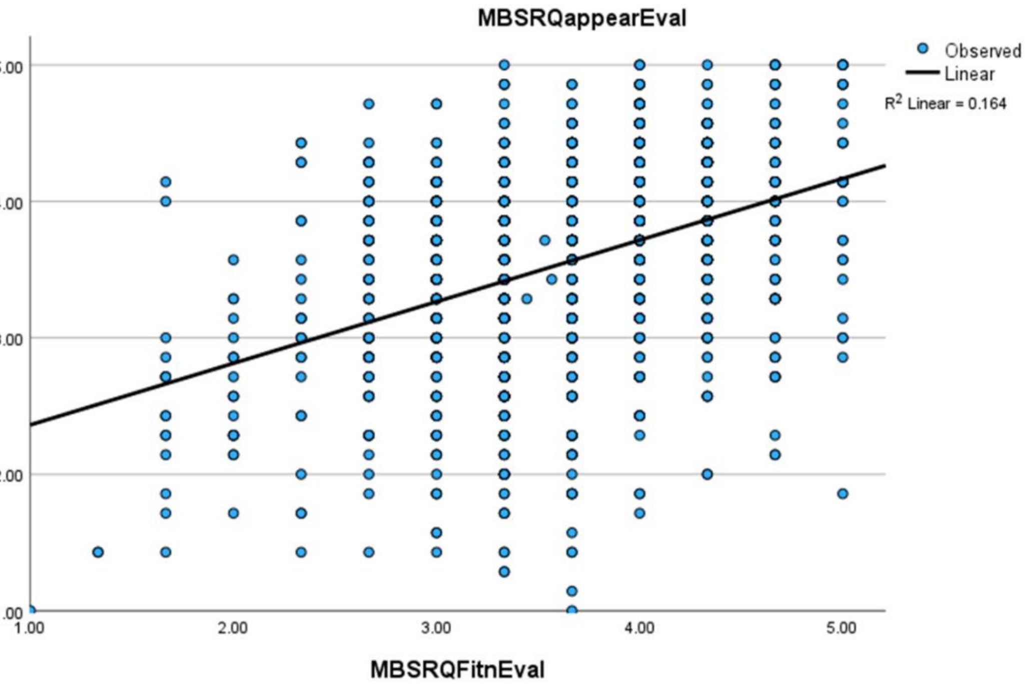
MBSRQappearEval
Observed
Linear
R2 Linear = 0.164
5.00
4.00
3.00
2.00
1.00
1.00
2.00
3.00
4.00
5.00
MBSRQFitnEval

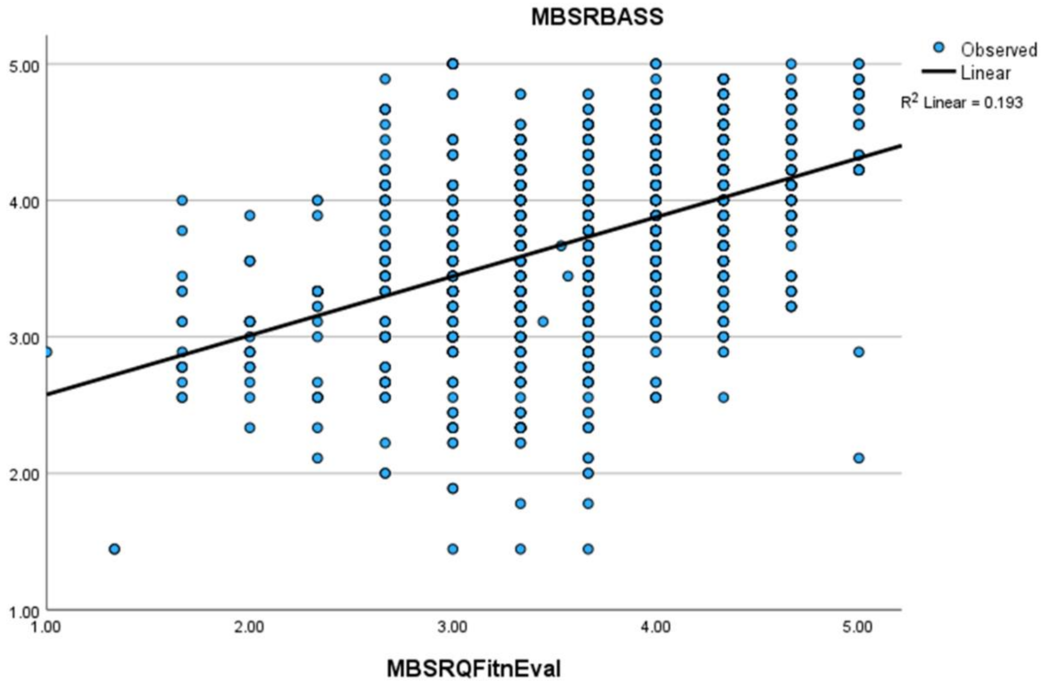
MBSRBASS
Observed
Linear
R2 Linear = 0.193
5.00
4.00
3.00
2.00
1.00
1.00
2.00
3.00
4.00
5.00
MBSRQFitnEval

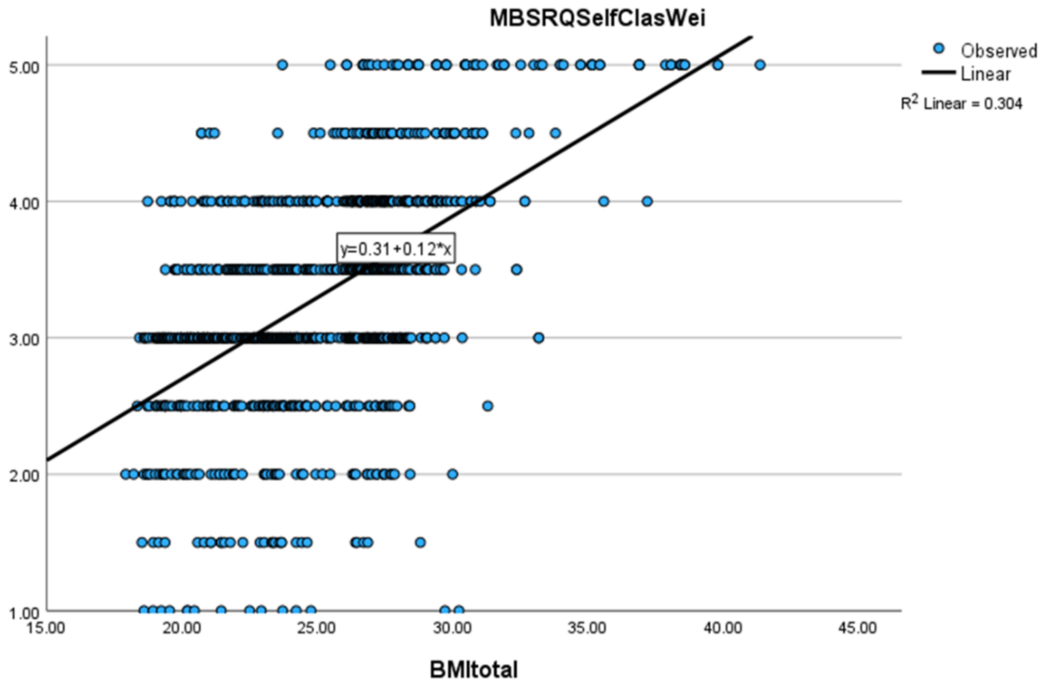
MBSRQSelfClasWei
Observed
Linear
R2 Linear = 0.304
y=0.31+0.12*x
5.00
4.00
3.00
2.00
1.00
15.00
20.00
25.00
30.00
35.00
40.00
45.00
BMItotal
